# Insights into Nutrient Contents, Fermentation Profiles, Bacterial Communities and Co-Occurrence Network of Small-Bale Oat Silage Prepared with/Without *Lentilactobacillus buchneri* or *Lacticaseibacillus rhamnosus*

**DOI:** 10.3390/microorganisms14010101

**Published:** 2026-01-02

**Authors:** Baiyila Wu, Xue Cao, Shuo Liu, Tong Ren, Yuxin Bao, Hua Mei, Shiba Liu, Chelegeri Zhao, Longli Cong, Shiyang Jiao, Huaxin Niu, Shubo Wen, Haifeng Wang, Yang Song

**Affiliations:** 1 College of Animal Science and Technology, Inner Mongolia Minzu University, Tongliao 028000, China; baiyilaw@imun.edu.cn (B.W.); xuecao0823@163.com (X.C.); liushy2025@163.com (S.L.); rentongxf@163.com (T.R.); meihua22@163.com (H.M.); niuhx@imun.edu.cn (H.N.); wen0516@126.com (S.W.); 2 Tongliao Academy of Agriculture and Animal Husbandry Sciences, Tongliao 028015, China; baoyx0211@163.com (Y.B.); longlicong168@163.com (L.C.); jsy5214863@163.com (S.J.); 3 Tongliao Agriculture and Animal Husbandry Development Center, Tongliao 028000, China; liushiba156@163.com (S.L.); chelegeri@163.com (C.Z.)

**Keywords:** small-bale, *Lacticaseibacillus rhamnosus*, *Lentilactobacillus buchneri*, oat silage, bacterial community structure

## Abstract

Oat is a forage with high protein value (10–14% DM) and good palatability, and is considered one of the main feed sources for ruminants. In this experiment, *Lacticaseibacillus rhamnosus* and *Lentilactobacillus buchneri* were selected as silage additives to investigate the fermentation quality, nutrient composition, microbial community and relationship between fermentation products and bacterial community of small-bale oat silage after ensiling. The experiment was set up with three treatment groups and three replications in each group, which were the control (C) group, *L. rhamnosus* (LR) group and *L. buchneri* (LB) group, and oat silages were subjected to 10-day and 30-day storage periods. The results show that both LR and LB additions significantly increased water-soluble carbohydrate, crude protein, lactic acid, propionic acid and acetic acid contents, and decreased pH, butyric acid, acid detergent fiber, neutral detergent fiber, and ammonia nitrogen contents and yeast and enterobacteria numbers in small-bale oat silage, compared with the C group. The highest content of acetic acid and the lowest numbers of enterobacteria and yeast were found in the LB group after 30 days of fermentation. *Lentilactobacillus* and *Lacticaseibacillus* were the dominant genera in the LB and LR groups, regardless of fermentation time. *Lentilactobacillus* and *Lacticaseibacillus* were positively correlated with a correlation value of 0.9, but both were negatively correlated with *Bacillus*. *Lentilactobacillus* and *Lacticaseibacillus* were positively correlated with acetic and lactic acids, while pH and butyric acid were positively correlated with *Bacillus*. This experiment revealed that the addition of homofermentative and heterofermentative lactic acid bacteria enhanced the relative abundance of *Lentilactobacillus* and *Lacticaseibacillus*, reduced harmful microbes, and improved fermentation quality of small-bale oat silage.

## 1. Introduction

As the animal husbandry and feed industries have experienced rapid growth in recent years, the demand from ruminants for high-quality feed has been increasing year by year [1]. Oat plants belong to the genus *Avena* of the family Poaceae, and they are not only typical crops that can be used as both grains and feeds but are also an important forage resource. They have a high dry matter yield (6–8 tons/ha) and good palatability, and they are widely planted in northern, northwestern, and southwestern China [2]. China’s Inner Mongolia is the largest oat-growing region in the country, with an oat cultivation area exceeding 133,400 hectares [3]. It also boasts the highest oat yield among all oat-growing regions in China. Most of the harvested oats are used for making green hay [4]. However, ensiling, a traditional method for preserving fresh crops, is currently widely adopted across numerous countries around the world [5]. Therefore, making fresh oats into silage can reduce environmental dependence in forage preservation [6].

The moisture, water-soluble carbohydrate, and protein contents of fresh forage before ensiling have a significant impact on the silage quality [7]. It is difficult to achieve ideal silage because fresh oats have high protein (10–14% DM) and low water-soluble carbohydrate contents (3–6% DM) compared with other fresh forages such as whole-plant corn, sweet sorghum, and sugarcane tops [8]. Using appropriate additives is beneficial for promoting the silage fermentation process and improving the fermentation quality of silage. *Lacticaseibacillus rhamnosus* belongs to homofermentative lactic acid bacteria and demonstrates a strong capacity to withstand acidic conditions. Under anaerobic conditions, *L. rhamnosus* can convert the water-soluble carbohydrates present in forages into short-chain fatty acids, with the resultant pH reduction inhibiting spoilage microorganisms and preserving the nutrients in silage [9]. *Lentilactobacillus buchneri* can use glucose as a substrate to produce compounds such as acetic acid, ethanol, lactic acid, and carbon dioxide during the silage fermentation process [10]. Wu et al. [11] revealed that the inoculation of *L. buchneri* and *Lactiplantibacillus plantarum* to *Leymus chinensis* silage not only increases the fermentation profile but also improves the aerobic stability. However, the knowledge on the characteristics and functions of *L. buchneri* and *L. rhamnosus* isolated from small-bale oat silage and their impacts on bacterial diversity and fermentation profiles of small-bale oat silage is lacking. Hence, we have isolated and identified *L. rhamnosus* and *L. buchneri* strains from small-bale oat silage using 16S ribosomal RNA gene sequencing and phenotyping. *L. rhamnosus* and *L. buchneri* not only have high acid resistance but also are resistant to low temperatures. It is imperative to explore shifts in fermentation profiles and bacterial diversities during the fermentation period when *L. rhamnosus* and *L. buchneri* are added to small-bale oat silage.

Even though some studies have investigated the fermentation profiles, bacterial diversities, and cellulose digestion of small-bale oat silage [12,13], only a small number have assessed the fermentation profiles and bacterial diversity of small-bale oat silage treated with/without *L. rhamnosus* or *L. buchneri*. Therefore, the impacts of *L. rhamnosus* and *L. buchneri* on the microbial population, bacterial diversity, and fermentation profile of small-bale oat silage were examined.

## 2. Materials and Methods

### 2.1. Lactic Acid Bacteria Strain Isolation

We have isolated several lactic acid bacteria strains with high fermentation profiles from oat (*Avena sativa*), *Caragana korshinskii*, alfalfa (*Medicago sative*), and *Leymus chinensis* (Chinese Rye Grass) silage. Ninety-six lactic acid bacteria strains were isolated from small-bale oat silage stored for 120 days according to different shapes and sizes. The isolation was performed using the dilution plate method: 10 g of silage sample was aseptically weighed and homogenized with 90 mL of 0.85% NaCl for 30 min, then serially diluted to 10^−6^. Subsequently, 0.2 mL of each dilution was spread on de Man, Rogosa and Sharpe agar plates, which were incubated for 48–72 h under 37 °C. Colonies with different shapes and sizes were picked individually and purified by streaking on fresh de Man, Rogosa and Sharpe agar plates 2–3 times to obtain pure LAB strains.

### 2.2. Ensiling Preparation and Lactic Acid Bacteria Inoculants

On 4 August 2024, we collected the early dough of grain-filling stage of oat (Helena) in the experimental field of Shuangxi Beef Cattle Breeding Farm (121°32′—122°45′ E, 44°24′—45°46′ N) from Zhurihe Town, Inner Mongolia. Forage (3600 kg) was harvested by machine and chopped to 15–25 mm chop length. Forage was wilted for 4 h, and a dry matter level of about 323 g/kg was obtained. The treatments included fresh forage without the application of inoculants (control; C), forage treated with 1 × 10^6^ CFU/g *L. rhamnosus* (LR), and forage treated with 1 × 10^6^ CFU/g *L. buchneri* (LB). For the treatment LR and treatment LB, *L. rhamnosus* and *L. buchneri* were cultured anaerobically in de Man, Rogosa and Sharpe broth at 30 °C for 96 h and 72 h, respectively, and subsequently diluted with 0.85% NaCl. Thereafter, *L. rhamnosus* and *L. buchneri* were added at a concentration of 1 × 10^6^ CFU/g, respectively. For treatment C, an equal volume of purified water was added. After fully mixing the LAB inoculant or purified water with the forage, each sample was wrapped tightly with six layers of plastic film via a bale wrapper (9YDB-55, Fangcheng Ltd., Qingdao, China), thereby creating an oxygen-free environment for the anaerobic fermentation of the round bales (60 cm length × 40 cm diameter), each of which measured the density with 660 kg/m^3^ and weighed approximately 200 kg. The bales were stacked in three layers for outdoor storage, with three replicates assigned to each treatment group, and oat silages were evaluated on days 10 and 30.

### 2.3. Fermentation Profile, Microbial Population, and Chemical Composition

Each silage sample (30 g) was homogenized in 270 mL of distilled water for 1 min, after which the mixture was filtered through a 0.22 μm membrane to obtain aqueous extracts. These extracts were subsequently used to quantify the concentrations of key organic acids (acetic, butyric, lactic, and propionic acids) and determine pH values, with pH measurements conducted using a glass electrode pH meter (PHS-320; Boqu Ltd., Shanghai, China). The ammonia nitrogen (NH_3_-N) content was analyzed via the phenol–hypochlorite method [14], while fermentation quality parameters were evaluated using ion-exclusion polymeric high-performance liquid chromatography (HPLC) equipped with a refractive index (RI) detector [15].

Under a sterile laminar flow hood, serial 10^−1^ to 10^−7^ dilutions were prepared to quantify the microbial populations. Mold and yeast were enumerated on potato dextrose agar (C020787, Tuopu Ltd., Shandong, China) adjusted to pH 3.4; enterobacteria on violet red bile agar (HB0134, Tuopu Ltd., Shandong, China); and lactic acid bacteria (LAB) on de Man, Rogosa, and Sharpe (MRS) agar (M1502; Tuopu Ltd., Shandong, China). Cultivation conditions varied by microbial group: LAB at 37 °C and both enterobacteria and mold/yeast at 30 °C [11]. Colony counts were derived from viable microbial assessments, with only plates containing 30–300 colonies selected for final enumeration.

Dry matter (DM) content measurements for fresh oat material and oat silage involved oven-drying the samples at 65 °C for 48 h, followed by pulverization through a 1 mm sieve using a Wiley mill (SM300, Retsch GmbH Ltd., Hann, Germany). The concentrations of neutral detergent fiber (NDF), water-soluble carbohydrates (WSCs), acid detergent fiber (ADF), and crude protein (CP) were quantified in accordance with the standard protocols described by Wu et al. [15] and Van Soest et al. [16].

### 2.4. Bacterial Sequencing Analysis

Forty grams of fresh oat material and silage was homogenized with one hundred sixty milliliters of sterile phosphate-buffered saline (PBS, pH 7.6), followed by shaking at 130 rpm for 3 h on an electronic signal oscillator. The mixture was then filtered through two layers of gauze, and the resulting filtrate was centrifuged at 4 °C and 11,000× *g* for 20 min. The supernatant was discarded, with the resultant pellets being preserved on dry ice immediately thereafter. For the amplification of the V3-V4 hypervariable regions of the 16S rDNA gene, the primer pair 341F (CCTACGGGNGCWGCAG) and 805R (GACTACHVGGGTATCTAATCC) was utilized. A 25 μL PCR reaction system was prepared, comprising 12.5 μL PCR premix, 25 ng template DNA, each primer at a volume of 2.5 μL, and PCR-grade water to make up the capacity. Post-amplification, PCR products were purified using PCR AMPure XT beads (Beckman Coulter Genomics, Danvers, MA, USA) and quantified with a Qubit fluorometer (Invitrogen, Carlsbad, CA, USA). Subsequent data processing and Illumina MiSeq sequencing were performed: A 97% similarity threshold was applied for the clustering of operational taxonomic units, which was implemented using UPARSE v7.1 [17]. After eliminating chimeric sequences, feature OTU sequences underwent taxonomic annotation at a confidence threshold of 0.7, with the Silva v138 16S rRNA database as the reference and the Ribosomal Database Project (RDP) classifier (v2.2) as the analytical tool [18]. Raw sequence data were ultimately deposited in the NCBI Sequence Read Archive under the accession number PRJNA1283064).

### 2.5. Statistical Analyses

The software JMP (version 15.2.0; SAS Institute, Tokyo, Japan) was used to analyze the impacts of fermentation duration and additive supplementation on microbial composition, bacterial diversity, and fermentation profile of oat silage. Two-way and one-way analyses of variance (ANOVAs) were applied to assess the impacts of fermentation time and inoculation, respectively, with multiple comparisons performed using Tukey’s test and statistical significance defined as *p* < 0.05. Additionally, microbial diversity, correlations between bacterial diversities and fermentation profiles, and bacterial co-occurrence networks were analyzed through Biomarker Cloud (https://www.biocloud.net/), a free online biomarker analysis platform (accessed on 20 November 2015).

## 3. Results and Discussion

### 3.1. Microbial Population and Chemical Composition of Fresh Oat

The microbial population and chemical composition analysis of fresh oats is shown in Table 1. The dry matter content of fresh oat in this study is 323 g/kg, which falls within the recommended range; thus, the fermentation quality is expected to be good. Gomes et al. [19] found that the fermentation quality would be greater for forages with ≥300 g/kg DM since the recommended range of DM for ensiling is 30–40%. The CP content of fresh oat forage was 92.6 g/kg and was relatively lower than the CP content of some other forages such as clover and alfalfa at their respective optimal harvesting stages [20]. The numbers of LAB were 3.93 log cfu/g, while the numbers of yeast and enterobacteria were 5.28 and 5.34 log cfu/g, which were much higher than the LAB number. Cai et al. [21] found that fermentation quality was higher when the LAB number of pre-ensiling crops exceeded 5.00 log cfu/g. This suggests that in the absence of silage inoculation, the harmful microorganisms attached to fresh oats may have an adverse impact during the fermentation process, leading to a loss of nutrients in silage. Therefore, in this experiment, adding different types of LAB at 6.00 log cfu/g to fresh forage can not only improve their nutritional value but also effectively inhibit the growth and reproduction of yeast, molds, and enterobacteria.

### 3.2. Fermentation Profile and Microbial Population

The impacts of distinct LAB strains on the fermentation profiles and bacterial community composition of small-bale oat silage are presented in Table 2. After 10 and 30 days of fermentation, The counts of LAB in LB and LR were significantly higher (*p* < 0.01) than those in C, which aligns with the finding that the pH reduction induced by LAB inoculants in silage suppressed the proliferation of spoilage microorganisms including enterobacteria and yeasts [11]. pH is a critical measure of fermentation efficiency, with a pH below 4.0 generally considered satisfactory for small grain cereal silages [22]. In this experiment, relative to C, the pH values of LB and LR were significantly reduced, with LR30 having the lowest pH value of 3.82. After 10 days of fermentation, organic acid contents were higher in LR10 and LB10 compared with C10. LR10 had the highest lactic acid content, while LB10 had the highest propionic acid and acetic acid contents. Acetic acid is one of the final products of *L. buchneri*, and it is also a key substance in maintaining the aerobic stability of silage [23]. The acetic acid content increased with extended fermentation time and was the highest in LB30 at 8.38 g/kg DM at the 30-day fermentation stage of silage. The effect of preservation period on acetic acid is not significant according to Table 2; it shows a numerical improvement, but it does not seem to be statistically different. Elevated acetic acid levels observed throughout silage fermentation could be linked to the presence of *L. buchneri*. *L. buchneri* can not only produce lactic acid by degrading WSCs but can also generate a large amount of acetic acid [10]. In addition, Bai et al. [24] demonstrated that *L. buchneri* is capable of converting lactic acid into acetic acid under specific conditions. Propionic acid is an important indicator for evaluating silage quality, and propionic acid contents were increased with extended fermentation time. After 30 days of ensiling, LR30 had the highest propionic acid of 4.47 g/kg DM. This indicates that Under anaerobic conditions, LAB can stimulate propionic acid bacteria to synthesize substantial amounts of propionic acid, thereby inhibiting the proliferation of detrimental microorganisms including enterobacteria and yeasts. Butyric acid bacteria can decompose crude protein to produce butyric acid, leading to nutrient loss in the silage. After 10-day and 30-day storage, the butyric acid content of both LB and LR was significantly (*p* < 0.01) lower than that of C, which might be more related to the acidic environment formed by the addition of LAB. The numbers of enterobacteria and yeast in LB and LR were also significantly reduced, which was attributed to the production of organic acids with antimicrobial activity during the fermentation process. Further research is needed to investigate the effects of the combined addition of *L. rhamnosus* and *L. buchneri* on the fermentation profile and microbial population of small-bale oat silage under the production conditions.

### 3.3. Nutrient Composition

The impacts of different types of LAB on the nutrient profile of small-bale oat silage are shown in Table 3. After 10-day and 30-day storage, the WSC amount significantly (*p* < 0.01) increased in both LB and LR, with the highest content of 49.68 g/kg DM observed in LR30. Tahir et al. [25] showed that the WSC content was reduced by the addition of LR to oat silage, which is different from the results of the present experiment. These results indicate significant differences in the degradation ability of WSCs between different *L. rhamnosus* strains. Addah et al. [26] discovered that adding LAB decomposes some complex carbohydrates (such as starch and fiber) into WSCs during the ensiling of whole-crop cereal forages. We have repeated the detection of WSCs, and the results were consistent. Hence, we further aim to study the effects of residual WSCs in silage on rumen fermentation and rumen microorganisms of beef cattle. Compared with C, the CP content in LR and LB was higher, but the ammonia nitrogen content was lower. This may be due to the presence of butyric acid bacteria in C, which converts CP into ammonia nitrogen. This result indicated that LAB rapidly produce acid to establish a stable acidic environment, thereby reducing the degradation and loss of protein at the source. On the other hand, LAB may promote other microorganisms to synthesize new crude proteins. ADF and NDF are important indicators for evaluating the nutritional profile of silage. The amounts of ADF and NDF in LR and LB were significantly (*p* < 0.01) reduced in this experiment. Analyzing the results, a reduction in NDF in the treated silages can be observed, which may partially explain the increase in residual WSCs. However, the residual WSC values remain high, which limits the biological explanation for this process. It is possible that the rate at which microorganisms decompose WSCs is slower than the rate at which they produce WSCs. The main components of ADF and NDF are cellulose and hemicellulose, and homofermentative and heterofermentative lactic acid bacteria under anaerobic conditions produce a series of enzymes (cellulase and hemicellulase), which are able to decompose cellulose and hemicellulose in the plant cell wall. In addition, the acidic environment produced by LAB fermentation may also cause certain damage to the structure of plant cell walls [13]. Results obtained from the present experiment were comparable to the observations of Desta et al. [27], where these authors reported that organic acid concentrations increased in Napier grass ensiled with formic acid, molasses, and a fibrolytic enzyme.

### 3.4. Bacterial Diversity and Bacterial Community Structure

**Figure 1:**
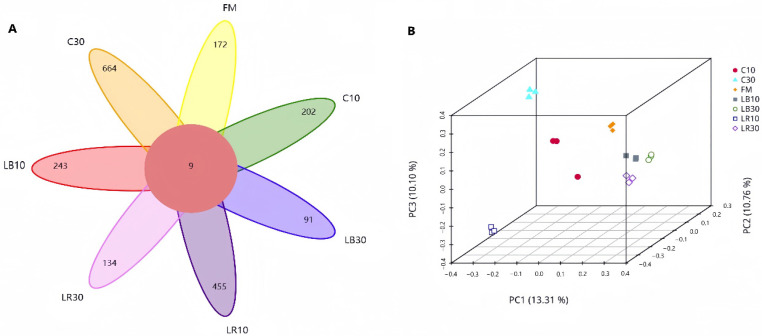
(**A**) Venn diagram illustrating the distribution of operational taxonomic units (OTUs) between fresh oat samples and small-bale silage. (**B**) Spatial distribution characterization of operational taxonomic units (OTUs) via principal component analysis in fresh oat forage and small-bale oat silage. C, control group; FM, fresh material; LR, *Lacticaseibacillus rhamnosus* group; LB, *Lentilactobacillus buchneri* group; The numbers following these group labels indicate the fermentation times.

The Venn diagram (Figure 1A) shows the counts of common and exclusive bacterial OTUs between fresh forage, C, LR, and LB. The results showed that 9 common bacterial OTUs and 1961 unique bacterial OTUs were detected. The quantities of unique bacterial OTUs in different fermentation stages were: 172 for fresh material, 900 for the 10-day fermentation, and 889 for the 30-day fermentation. Bacterial community structure of oat silages was evaluated via principal component analysis (PCA), with the results presented in Figure 1B. The results showed obvious separation and differences in bacterial communities between fresh oat and small-bale oat silage because bacterial community structures attached to the surfaces of fresh oat and small-bale oat silage were different. LR30 and LB30 are close in distance, but they deviate from C30, which indicated that adding homofermentative and heterofermentative LAB to oat silage can alter the bacterial community structure.

**Figure 2:**
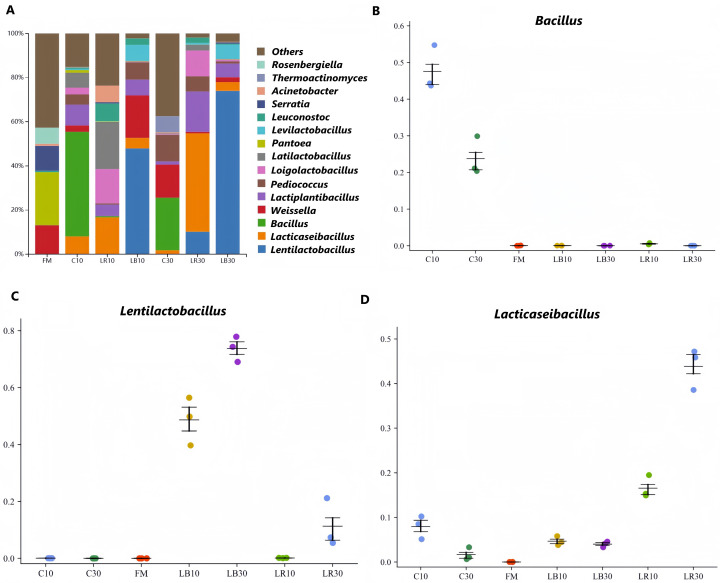
Bacterial community structure at the genus level (**A**) and error bar plots (**B**–**D**) representing fresh oat material and small-bale oat silage. C, control group; FM, fresh material; LR, *Lacticaseibacillus rhamnosus* group; LB, *Lentilactobacillus buchneri* group; The numbers following these group labels indicate the fermentation times.

The genus level of the bacterial composition in raw material and oat silage is shown in Figure 2A. *Rosenbergiella* and *Pantoea* are the most common genera in fresh oats, and they are frequently detected in other forages such as *Caragana korshinskii* and alfalfa [28,29]. The action of these microorganisms is similar to that of *Bacillus*, competing with LAB for carbohydrate resources, thereby leading to nutrient loss [30]. By extending the storage time from 10 to 30 days, the relative abundance of *Bacillus* in C gradually decreased from 47% to 24% (Figure 2B). This could be attributed to the fact that the LAB in oat silage inhibited the growth of *Bacillus*, leading to a rapid decrease in its relative abundance. LAB played diverse roles in silage fermentation, and were able to reduce pH by producing lactic acid, providing an acidic environment and inhibiting the growth and reproduction of detrimental microorganisms. In this experiment, *Lentilactobacillus* and *Lacticaseibacillus* were the predominant bacterial genera in LB and LR, respectively. Abundance proportion of *Lacticaseibacillus* in LR increased from 17% to 45%, extending storage time from 10 to 30 days (Figure 2D). This may be related to the ability of *L. rhamnosus* to produce large amounts of lactic acid, lowering the pH of the oat silage. The most significant increase in the relative abundance of *Lentilactobacillus* was observed in LB, which increased from 48% to 74% with the extension of fermentation time (Figure 2C). Interestingly, *L. buchneri* was known to tolerate acidic conditions and can metabolize lactic acid to produce acetic acid, enhancing its survival throughout fermentation [23]. In addition, *Weissella* can also convert WSCs to produce acetic acid [21]. The relative abundance of *Weissella* in LB10 was 19%, which can explain why LB had the highest acetic acid content. Next-generation sequencing can only detect the genus level of microorganisms in oat silage. Hence, more in-depth studies are required to clarify the linkages between metabolic products and microbial populations in LAB-treated oat silage through gas chromatography–mass spectrometry and single-molecule real-time sequencing techniques.

### 3.5. Co-Occurrence Networks of Bacterial Community

**Figure 3:**
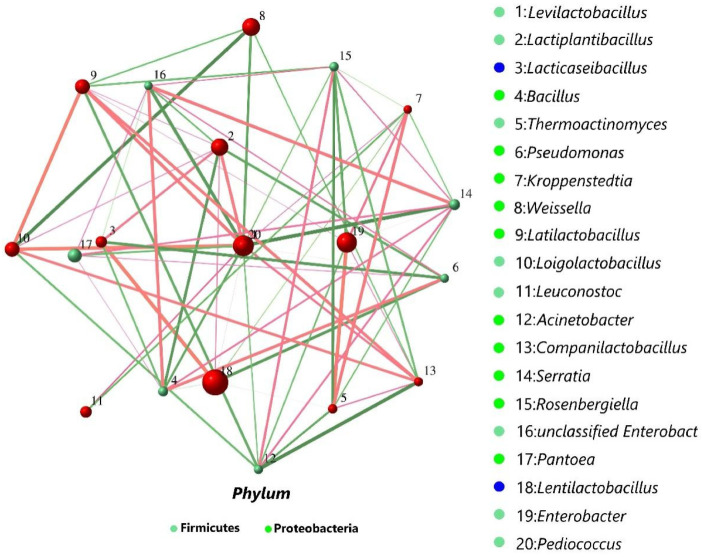
Co-occurrence networks depicting the genus-level bacterial community structure; each circle denotes a microbial genus, and circle size is indicative of its relative abundance. Lines illustrate intergeneric correlations: line thickness indicates correlation strength, and positive correlations are represented by red hues, while green hues indicate negative correlations.

The co-occurrence network of bacterial community in small-bale oat silage is illustrated in Figure 3. A strong positive correlation was observed between *Lentilactobacillus* and *Lacticaseibacillus*, with a correlation coefficient of 0.9. *Lentilactobacillus* and *Lacticaseibacillus* were the important genera in LB and LR, which rapidly reduced the pH, and they may produce some antimicrobial substances that have an inhibitory effect on harmful microorganisms [21]. Therefore, *Bacillus* was negatively correlated with *Lentilactobacillus* and *Lacticaseibacillus*, with correlation values of −0.61 and −0.45, respectively. Furthermore, Bacillus was positively correlated with *Pantoea* and *Serratia*, yielding a correlation values of 0.48 and 0.59. This may be because during the silage fermentation process, they are all capable of decomposing the protein in the silage, generating alkaline substances that consume lactic acid, thus causing the pH value to rise [11]. They are harmful microorganisms present in silage that affect the quality and nutritional value of silage.

### 3.6. Correlation Analysis Between the Bacterial Community with Fermentation Products and pH

**Figure 4:**
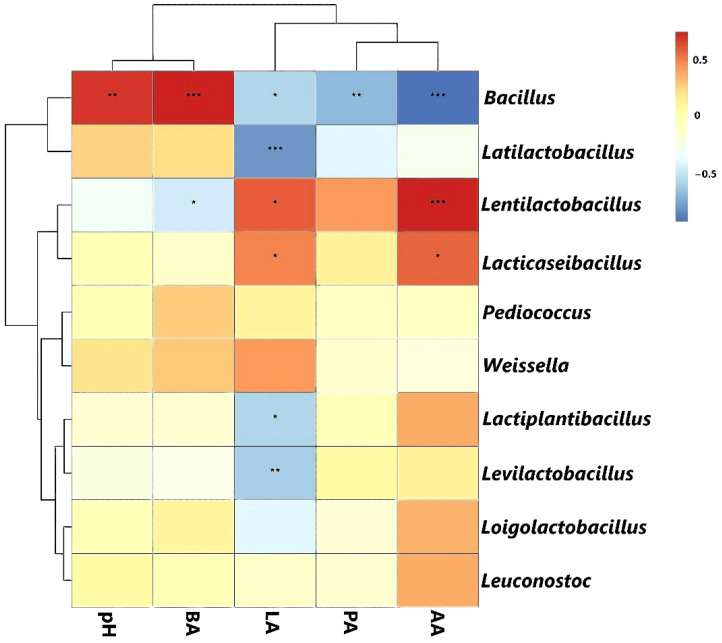
Correlations between the bacterial community and main fermentation parameters (pH, lactic acid [LA], propanoic acid [PA], butyric acid [BA], and acetic acid [AA]) were analyzed. Statistical significance is denoted as: * (0.01 < *p* < 0.05), *** (*p* < 0.001), and ** (0.001 < *p* < 0.01).

Silage is preserved using anaerobic fermentation, and its fermentation quality is highly dependent on the stability of the bacterial community. In this study, *Lentilactobacillus* and *Lacticaseibacillus*, as the dominant bacteria present during oat silage fermentation, were able to inhibit the growth of harmful microorganisms. *L. rhamnosus*, *Lactiplantibacillus plantarum*, and *Lactococcus lactis* are homofermentative LAB that can rapidly acidify and convert glucose to organic acids, mainly lactic acid during fermentation, significantly lowering the pH and rapidly establishing an acidic environment [31]. *L. buchneri* and *Weissella cibaria* are heterofermentative LAB, whose metabolites include not only acetic acid but also lactic acid, ethanol, carbon dioxide, and 1,2-propanediol [32]. *Bacillus* is a harmful bacterium that competed with LAB for WSCs in the early stage of oat silage fermentation, inhibiting the acidification process dominated by LAB, thus leading to the spoilage of silage [33]. Spearman’s rank correlation analyses between the main bacteria and fermentation parameters after 10 and 30 days of fermentation are shown in Figure 4. The genera *Lentilactobacillus* and *Lacticaseibacillus* were positively correlated (*p* < 0.05) with acetic and lactic acid amounts. In contrast, *Latilactobacillus*, *Lactiplantibacillus*, and *Levilactobacillus* were negatively correlated (*p* < 0.05) with lactic acid levels. *Bacillus* showed significant positive correlations (*p* < 0.01) with pH (r = 0.68) and butyric acid (r = 0.73) and negative correlations (*p* < 0.05) with lactic (r = −0.56), propionic (r = −0.68), and acetic acids (r = −0.93). In summary, although the bacterial community structure was complex during the fermentation process, specific associations were evident between volatile fatty acids and the lactic acid bacteria and *Bacillus* present in small-bale oat silage.

## 4. Conclusions

*L. rhamnosus* and *L. buchneri* inoculations enhance the fermentation profile of small-bale oat silage, minimize dry mater loss, increase the abundance proportion of *Lentilactobacillus* and *Lacticaseibacillus*, and decrease the abundance proportion of *Bacillus*. These inoculations resulted in a substantial increase in nutritional components and short-chain fatty acids while effectively reducing the proliferation rate of yeast and enterobacteria. Therefore, the inoculation of *L. rhamnosus* and *L. buchneri* had a positive effect on fermentation products, nutrient composition and microbial diversity of oat silage, and these results provide a theoretical foundation for promoting the growth of beneficial LAB and inhibiting undesirable microorganisms.

## Figures and Tables

**Table 1:** Chemical and microbial compositions of pre-ensiling oat.

	Frash Oat
Dry matter (g/kg)	323 ± 5.18
pH	5.68 ± 0.34
Crude protein (g/kg DM)	92.64 ± 1.47
Neutral detergent fiber (g/kg DM)	378.56 ± 21.67
Acid detergent fiber (g/kg DM)	207.76 ± 3.46
Water-soluble carbohydrate (g/kg DM)	41.32 ± 0.65
Ammonium nitrogen (g/kg DM)	2.04 ± 0.21
Lactic acid bacteria (log cfu/g)	3.93 ± 0.54
Yeasts (log cfu/g)	5.28 ± 0.27
Enterobacteria (log cfu/g)	5.34 ± 0.43

Data are mean of duplicate analyses.

**Table 2:** Fermentation characteristics and microbial populations of small-bale oat silage prepared with/without *Lentilactobacillus buchneri* or *Lacticaseibacillus rhamnosus*.

	10 Days				30 Days				2-Way ANOVA		
	C	LR	LB	SE	C	LR	LB	SE	I	S	I × S
Dry matter (g/kg)	328	331	334	5.23	336	341	429	6.99	NS	NS	NS
pH	5.38 a	4.56 c	4.89 b	0.17	4.07 A	3.82 B	3.93 B	0.26	**	**	*
Lactic acid (g/kg DM)	8.38 c	14.67 a	12.24 b	0.45	18.57 B	23.29 A	24.47 A	1.23	**	**	**
Acetic acid (g/kg DM)	3.33 c	4.66 b	6.69 a	0.32	5.22 B	4.64 C	8.38 A	0.28	**	NS	**
Propionic acid (g/kg DM)	2.55 b	3.79 a	4.29 a	0.82	3.24 B	4.47 A	4.38 A	0.65	**	**	NS
Butyric acid (g/kg DM)	3.41 a	1.78 c	2.67 b	0.14	1.75 A	0.26 B	0.28 B	0.26	**	**	**
Lactic acid bacteria (log cfu/g)	6.42 b	8.46 a	8.56 a	0.79	7.34 B	8.46 A	8.54 A	0.66	**	**	**
Yeasts (log cfu/g)	7.42 a	4.63 b	3.29 c	0.73	8.58 A	4.24 B	2.96 C	0.38	**	**	**
Enterobacteria (log cfu/g)	7.23 a	4.87 b	3.74 c	0.68	6.63 A	4.26 B	2.78 C	0.59	**	**	**

Values represent the means of triplicate silage samples. For the same preservation period, values labeled with distinct lowercase (a–c) or uppercase (A–C) letters denote significant differences (*p* < 0.05). The terms I, S, and I × S correspond to the effects of inoculation treatments, preservation durations, and their interaction, respectively. Statistical significance is indicated by ** (*p* < 0.01), * (*p* < 0.05), and NS (*p* ≥ 0.05). SE refers to the standard error of the mean.

**Table 3:** Nutrient composition of small-bale oat silage prepared with/without *Lentilactobacillus buchneri* or *Lacticaseibacillus rhamnosus*.

	10 Days				30 Days				2-Way ANOVA		
	C	LR	LB	SE	C	LR	LB	SE	I	S	I × S
Water-soluble carbohydrates (g/kg DM)	42.83 b	46.37 a	47.83 a	2.28	46.23 B	49.68 A	48.71 A	2.02	**	**	*
Acid detergent fiber (g/kg DM)	206.37 a	186.13 b	197.76 b	4.28	198.63 A	173.82 B	176.49 B	5.09	**	**	**
Neutral detergent fiber (g/kg DM)	387.27 a	364.62 b	351.48 c	7.64	346.62 A	326.27 B	310.18 C	8.09	**	*	*
Crude protein (g/kg DM)	100.31 c	110.52 b	120.86 a	3.27	100.03 B	110.82 A	120.26 A	4.59	**	NS	**
Ammonium nitrogen (g/kg DM)	2.67 a	1.27 c	1.63 b	0.13	3.18 A	1.82 C	2.18 B	0.22	**	*	NS

Values represent the means of triplicate silage samples. For the same preservation period, values labeled with distinct lowercase (a–c) or uppercase (A–C) letters denote significant differences (*p* < 0.05). The terms I, S, and I × S correspond to the effects of inoculation treatments, preservation durations, and their interaction, respectively. Statistical significance is indicated by ** (*p* < 0.01), * (*p* < 0.05), and NS (*p* ≥ 0.05). SE refers to the standard error of the mean.

## Data Availability

The original contributions presented in this study are included in the article. Further inquiries can be directed to the corresponding authors.
